# Phosphorylated Osteopontin Secreted from Cancer Cells Induces Cancer Cell Motility

**DOI:** 10.3390/biom11091323

**Published:** 2021-09-07

**Authors:** Yoshinobu Kariya, Midori Oyama, Yukiko Kariya, Yasuhiro Hashimoto

**Affiliations:** Department of Biochemistry, Fukushima Medical University School of Medicine, Fukushima 960-1295, Japan; oyamami@josai.ac.jp (M.O.); ykk-kari@fmu.ac.jp (Y.K.); yasuc@fmu.ac.jp (Y.H.)

**Keywords:** osteopontin, phosphorylation, cancer, migration

## Abstract

Osteopontin (OPN) plays a pivotal role in cancer cell invasion and metastasis. Although OPN has a large number of phosphorylation sites, the functional significance of OPN phosphorylation in cancer cell motility remains unclear. In this study, we attempted to investigate whether phosphorylated OPN secreted from cancer cells affect cancer cell migration. Quantitative PCR and Western blot analyses revealed that MDA-MB435S, A549, and H460 cells highly expressed OPN, whereas the OPN expression levels in H358, MIAPaca-2, and Panc-1 cells were quite low or were not detected. Compared with the cancer cell lines with a low OPN expression, the high OPN-expressing cancer cell lines displayed a higher cell migration, and the cell migration was suppressed by the anti-OPN antibody. This was confirmed by the OPN overexpression in H358 cancer cells with a low endogenous OPN. Phos-tag ELISA showed that phosphorylated OPN was abundant in the cell culture media of A549 and H460 cells, but not in those of MDA-MB435S cells. Moreover, the A549 and H460 cell culture media, as well as the MDA-MB435S cell culture media with a kinase treatment increased cancer cell motility, both of which were abrogated by phosphatase treatment or anti-OPN antibodies. These results suggest that phosphorylated OPN secreted from cancer cells regulates cancer cell motility.

## 1. Introduction

Protein phosphorylation is a major posttranslational modification occurring on more than half of all human proteins [1], and plays a key regulatory role in signal transduction and metabolism [2]. Over the last decade, most studies of protein phosphorylation have focused exclusively on the cytoplasmic and nuclear proteins, but not on extracellular matrix (ECM) proteins, because of the limited availability of ECM proteins. Recent advances in mass spectrometry, however, have enabled sensitive analyses of the phosphorylation of ECM proteins [3,4]. In addition, the discovery of Golgi-casein kinases responsible for secreted proteins increased attention to extracellular phosphoprotein [5,6]. Thus, many phosphorylation sites of ECM proteins have been identified in the analyses using cell culture, tissue, and body fluid samples.

Analyses of body fluids have shown that extracellular phosphoproteins are particularly abundant in samples from cancer patients compared with those from healthy patients [4,7]. Furthermore, the analyses of the extracellular phosphoproteins secreted from basal and luminal type breast cancer cell lines and the phosphopeptides obtained from breast cancer patient plasma have demonstrated the presence of breast cancer type-specific phosphorylation sites in ECM proteins [8]. Considering the fundamental roles of ECM proteins in cancer cell adhesion, migration, survival, and stemness, understanding the relationship between the phosphorylation and functional activities in ECM proteins would provide a novel regulatory mechanism of cancer progression.

One of the ECM proteins, osteopontin (OPN; also known as Spp1), was originally identified as a secreted phosphoprotein associated with transformation [9,10,11]. OPN is highly expressed in different types of cancers, such as colorectal, gastric, pancreatic, liver, breast, lung, and prostate cancer, as well as melanoma, glioblastoma, and sarcoma [12,13]. The elevated OPN levels in the tumor tissues and plasma are correlated with a poor prognosis and reduced survival in cancer patients [14]. Recent studies have shown that OPN promotes tumorigenesis and metastasis by regulating cancer cell adhesion, migration, invasion, and proliferation through the interaction with integrins and CD44 [15]. Thus, OPN plays key roles in tumor development and cancer progression.

OPN has the largest proportion of phosphorylated sites [4] and past studies have identified more than 36 phosphorylation sites within the OPN molecule [4,16,17,18,19]. OPN is phosphorylated by Golgi-casein kinases such as FAM20C and VLK [4,5,20,21], and the OPN phosphorylation levels and sites are influenced by the *O*-glycosylation status [16,19] and 1,25-(OH)_2_D_3_ [22]. The phosphorylation state of OPN affects cell adhesion and interactions with other proteins [16,19,23,24,25,26,27]. In addition, highly phosphorylated human milk OPN promotes cell migration in human placenta choriocarcinoma cell lines, whereas less phosphorylated OPN fails to stimulate cell migration [28]. These past studies used purified OPNs derived from non-cancerous tissues or a recombinant OPN for their functional assays. Therefore, the role of phosphorylated OPN secreted from cancer cells in cancer cell behavior remains unclear. Considering the pivotal role of OPN in cancer progression, an understanding of the functional regulation of OPN by phosphorylation in cancer is important in order to overcome cancer and to develop novel cancer therapies. Thus, we tried to examine the relationship between phosphorylated OPN derived from cancer cells and cancer cell migration.

Here, we show that cancer cell motility is positively associated with the phosphorylation level of OPN in the conditioned culture media (CM) of cancer cells. CM containing phosphorylated OPN promoted cell migration, which was inhibited by phosphatase treatment or the OPN function blocking antibody.

## 2. Materials and Methods

### 2.1. Cell Culture

HEK293T (human embryonic kidney), A549 (human lung cancer), MIAPaca-2 (human pancreatic cancer), and Panc-1 (human pancreatic cancer) cell lines were provided by RIKEN BRC through the National Bio-Resource Project of the MEXT, Japan. H358 (human lung cancer), H460 (human lung cancer), and MDA-MB435S (human melanoma) cell lines were obtained from ATCC. Panc-1, H358, and H460 cell lines were cultured in RPMI-1640 (WAKO, Osaka, Japan, #189-02025) supplemented with 1 mM sodium pyruvate (WAKO, #190-14881), 2.5 g/L D (+)-glucose (WAKO, #079-05511), 10 mM HEPES, 10% FBS, and penicillin-streptomycin sulfate (WAKO, #168-23191). Other cell lines were cultured in DMEM (WAKO, #043-30085) supplemented with 10% FBS, and in penicillin-streptomycin sulfate (WAKO, #168-23191). These cell lines were maintained in low passage cultures.

### 2.2. Quantitative PCR (qPCR)

The total RNA of human normal adult lungs was purchased from BioChain (Newark, CA, USA, #R1234152-50). Cancer cell lines were plated and the total RNA was isolated independently using NucleoSpin RNA Plus (MACHEREY-NAGEL, Dueren, Germany, #740984.50). cDNA synthesis was performed using Prime Script RT Master Mix (Takara, Shiga, Japan, #RR036A). qPCR was performed using the PrimeTime Gene Expression Master Mix (IDT, San Jose, CA, USA, #1055772) and PrimeTime Mini qPCR Assay (IDT) with a StepOnePlus machine (Applied Biosystems). The values were calculated using HPRT1 gene transcription as a reference for normalization. For qPCR, the following primers were used:

SPP1-Fwd: 5′-CCCCACAGTAGACACATATGATG-3′;
SPP1-Rev: 5′-TTCAACTCCTCGCTTTCCAT-3′;
HPRT1-Fwd: 5′-TTGTTGTAGGATATGCCCTTGA-3′;
HPRT1-Rev: 5′-GCGATGTCAATAGGACTCCAG-3′.

### 2.3. Western Blot

The proteins resolved by SDS-PAGE were transferred to nitrocellulose membranes. After blocking with 5% skim milk, the membranes were probed with a rabbit polyclonal antibody against OPN (O-17, IBL, Gunma, Japan, #18625, 1 µg/mL). The membranes were washed three times with TBS containing 0.1% Tween-20 (T) for 5 min, and were incubated with an HRP-conjugated anti-rabbit IgG antibody (Promega, Madison, WI, USA, #W401B, 1:5000) or an HRP-conjugated phos-tag (WAKO, #301-93531). After washing three times with TBST for 5 min, the protein detection was completed using an ImmunoStar Zeta (WAKO, #297-72403) or a Trident femto-ECL reagent (GeneTex, Alton Pkwy Irvine, CA, USA, #GTX14698), and was imaged using Imager and Image Saver software (ATTO, Tokyo, Japan, #AE-9300H-CP).

### 2.4. Preparation of CM and Purification of Recombinant OPN

For the preparation of the CM, cells were grown to confluence in a growth medium, washed twice with PBS, and then incubated in a serum-free medium. After 2 days, the collected serum-free medium was centrifuged at 190× *g* for 10 min at 4 °C to remove the cell debris. The pre-cleared medium was buffer exchanged with PBS and then concentrated to 1% original volume using the Amicon Ultra-15 10K device (MerckMillipore, Millipore, CA, USA, #UFC901024).

For purification of the recombinant OPN, the proteins in the serum-free cell culture media of the OPN-HEK293T transfectant [16] were precipitated with 80% saturated ammonium sulfate and then the precipitate was dissolved in and dialyzed against an equilibration buffer (50 mM sodium phosphate, 300 mM NaCl, 0.1% CHAPS, and 0.005% Brij 35, pH 7.0) at 4 °C overnight. The dialyzed sample was applied to a TALON Metal affinity column (Takara, #635501). After washing with an equilibration buffer, the bound proteins were eluted with an equilibration buffer containing 150 mM imidazole. The fractions that contained the OPN were applied to a heparin sepharose column (GE Healthcare, Waltham, MA, USA, #17-0467-01) that had been pre-equilibrated with a heparin column equilibration buffer (10 mM sodium phosphate, 150 mM NaCl, 0.1% CHAPS, and 0.005% Brij 35, pH 7.0). After washing with a heparin column equilibration buffer, the bound proteins were eluted with a heparin column elution buffer (10 mM sodium phosphate, 300 mM or 500 mM NaCl, 0.1% CHAPS, and 0.005% Brij 35, pH 7.0). The eluted fractions that contained the OPN were dialyzed against a Nickel Magnetic Beads equilibration buffer (50 mM sodium phosphate, 300 mM NaCl, 10 mM imidazole, 0.1% CHAPS, and 0.005% Brij 35, pH 8.0). The samples were added to pre-equilibrated Nickel Magnetic Beads (MerckMillipore, #LSKMAGH02) with a Nickel Magnetic Beads equilibration buffer and were rotated at 4 °C for 3 h. The samples were placed in a Magnetic Beads stand, washed three times with the equilibration buffer, and then the bound proteins were eluted with elution buffer (50 mM sodium phosphate, 300 mM NaCl, 300 mM imidazole, 0.1% CHAPS, and 0.005% Brij 35, pH 8.0). The eluted samples were dialyzed against PBS containing 0.1% CHAPS and 0.005% Brij 35 and were used for the following assays.

### 2.5. Cell Migration and Invasion Assays

Cell migration and invasion assays were performed using Boyden chambers (BD Transduction Laboratories, Franklin Lakes, NJ, USA, cell culture companion plates #353504 and 8.0-µm inserts #352097). For the invasion assay, each well of the upper inserts was coated with 100 µL of Matrigel (BD Transduction Laboratories, #354234).

For the CM treatment, the CM samples (4 µL) were added to a cell suspension (1 × 10^5^ cells in 200 µL of serum-free medium) and were incubated for 20 min at room temperature.

For the inhibitory assay, 4 µL of CM or a recombinant OPN (300 ng) was treated with 0.4 µL of mouse monoclonal anti-OPN antibody (clone 53, Enzo Life Sciences, Plymouth Meeting, PA, USA, #ADI-905-629) for 20 min at room temperature. For the alkaline phosphatase treatment, 4 µL of CM was treated with 0.4 µL of calf intestine alkaline phosphatase (CIAP, TOYOBO, Osaka, Japan, #CAP-101, 5.2 U/reaction) in 6 µL of reaction buffer for 1 h at 37 °C. The samples were added to the cell suspension (1 × 10^5^ cells in 200 µL of serum-free medium) and were incubated for 20 min at room temperature.

For the phosphorylation of OPN, CM (4 µL) or a recombinant OPN (300 ng) was treated with 0.6 µL of recombinant human casein kinase II (Enzo Life Sciences, #BML-SE124-0010, 1000 U/reaction) for 2 h at 37 °C in a reaction buffer (20 mM HEPES, 15 mM NaCl, 12 mM MgCl_2_, 0.3 mM ATP, pH 7.5). The samples (6 µL) were added to a cell suspension (1 × 10^5^ cells in 200 µL of serum-free medium).

Cells (1 × 10^5^) in 200 µL of serum-free medium with or without sample were added to each well of the upper chambers, and 750 µL of growth medium was placed in the lower chamber. After 22 h, the cells were fixed with 2.5% glutaraldehyde for 10 min and were stained with 0.5% crystal violet in 20% methanol for 20 min. Matrigel and un-migrated cells were removed with cotton swabs. The cells were counted in three random fields for quantification.

### 2.6. Plasmids and Transfectants

To introduce V5-tag into human OPN complementary DNA (cDNA), stop codon of OPN cDNA in pENTR/D-TOPO (Thermo Fisher Scientific, Waltham, MA, USA, #K2400-20) [16] was mutated into alanine residue and then recombined to a pcDNA6.2/V5-DEST vector (Thermo Fisher Scientific, #12489-027) using the LR clonase II enzyme mix (Thermo Fisher Scientific, #11791). The plasmids were transfected into H358 cells using Lipofectamine 3000 (Invitrogen, Waltham, MA, USA, #L300001). To establish a stable cell line, the cells were selected with 10 µg/mL blasticidin S (Calbiochem, San Diego, CA, USA, #203350).

### 2.7. Phos-Tag Enzyme Linked Immunosorbent Assay (ELISA)

The wells of an ELISA plate (Thermo Fisher Scientific, #445101) were coated with 50 µL of 1 µg/mL anti-OPN antibody (O-17) in a 50 mM carbonate buffer (0.015 M Na_2_CO_3_, 0.035 M NaHCO_3_, pH 9.6) overnight at 4 °C. The wells were blocked with 200 µL of 1% BSA in TBS-T for 1 h at 37 °C, and then incubated with 100 µL of CM with or without CIAP or kinase treatment for 2 h at room temperature. The wells were then washed three times with TBS-T, followed by incubation for 1 h at room temperature with 50 µL of 1 µg/mL anti-OPN antibody (clone 53). After washing three times with TBS-T, the wells were further incubated with 50 µL of HRP-conjugated goat anti-mouse IgG antibody (Cell Signaling, Danvers, MA, USA, #7076, 1:5000). To detect the phosphorylated form of OPN, the wells of the ELISA plate were incubated with 50 µL of HRP-conjugated phos-tag solution instead of the anti-OPN antibody (clone 53) for 2 h at room temperature. After washing the wells four times with TBS-T, color development was obtained using the TMB microwell peroxidase substrate system (SeraCare, Milford, MA, USA, #50-76-11), and the reaction was stopped by phosphoric acid. The color intensity was measured at 450 nm using a microplate reader (BioRad, Hercules, CA, USA, model 680).

### 2.8. Statistics and Reproducibility

The results are presented as mean ± SEM of three independent experiments conducted in triplicate. Statistical comparisons were calculated between two groups using unpaired two-tailed Student’s *t*-test and among the groups using one-way ANOVA followed by a Tukey post-hoc test, with GraphPad Prism Version 5.0a and SPSS statistics 26 software. A *p* value of < 0.05 was considered to be significant. The Western blot results shown are representative images of three independent experiments, with similar results.

## 3. Results

### 3.1. OPN Expression in Cancer Cell Lines

**Figure 1:**
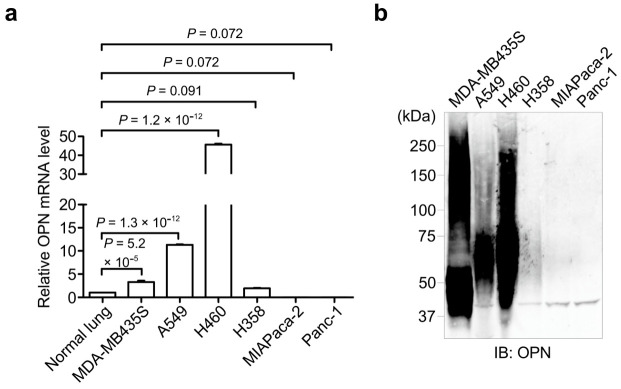
Analyses of the OPN expression in cancer cell lines. (**a**) Relative mRNA expression (qPCR) of OPN in cancer cell lines. The mRNA level of the normal lung was set to 1. One-way ANOVA and Tukey post-hoc test, mean ± SEM of three independent assays conducted in triplicate. (**b**) Western blot analysis of endogenous OPN levels in the culture conditioned media of various cancer cell lines. The unprocessed blot image is shown in Figure S1.

To analyze the OPN expression levels in cancer cell lines, we performed qPCR (Figure 1a) and Western blot (Figure 1b) analyses of the OPN expression in several cancer cell lines, including melanoma cell line MDA-MB435S; lung cancer cell lines A549, H460, and H358; and pancreatic cancer cell lines MIAPaca-2 and Panc-1. The qPCR analysis using specific primer sets to OPN revealed that OPN was highly expressed in MDA-MB435S, A549, and H460 cancer cells compared with normal lung cells, while the OPN expression levels in H358, MIAPaca-2, and Panc-1 cancer cells were low or not detected. In addition, Western blot using the anti-OPN antibody showed that highly secreted OPN was found in MDA-MB435S, A549, and H460 cells, but not in H358, MIAPaca-2, and Panc-1 cells.

### 3.2. Identification of OPN as a Cell Motility Inducing Factor in Culture Media of Cancer Cells

**Figure 2:**
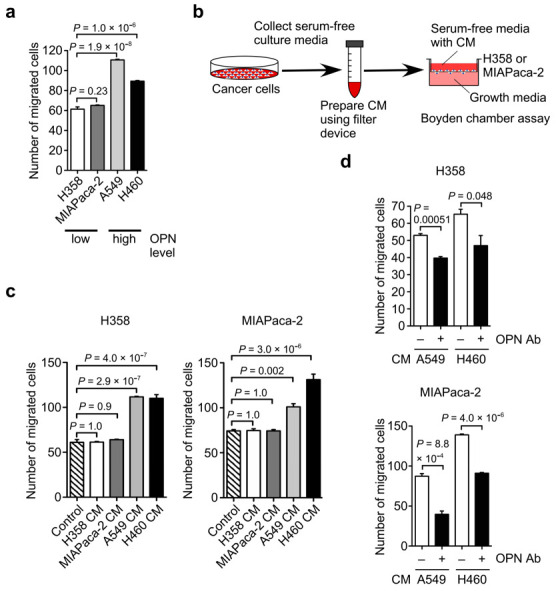
Effect of endogenous OPN secreted from cancer cells on cancer cell migration. (**a**) Migration activity of H358, MIAPaca-2, A549, and H460 cells. One-way ANOVA and Tukey post-hoc test, mean ± SEM of three independent assays conducted in triplicate. (**b**) Schematic procedure of the cell migration assay using conditioned culture media (CM) from cancer cells. (**c**) The effect of the CM from H358, MIAPaca-2, A549, and H460 cells on the cell migration of H358 and MIAPaca-2 cells. PBS was used as a sample instead of CM in the control experiments. One-way ANOVA and Tukey post-hoc test, mean ± SEM of three independent assays conducted in triplicate. (**d**) Effect of OPN function blocking antibody (OPN Ab) on the cell migration of H358 and MIAPaca-2 cells induced by the CM from A549 and H460 cells. – and + indicate CM without and with OPN Ab treatment, respectively. Two-tailed unpaired Student’s *t*-test, mean ± SEM of three independent assays conducted in triplicate.

To clarify the role of OPN in cancer cell migration, we first examined the relationship between OPN expression and cancer cell motility. Cell migration assay using the Boyden chamber demonstrated that A549 and H460 cells showed higher cell motility compared with H358 and MIAPaca-2 cells (Figure 2a). Considering the results of the OPN expression levels in Figure 1, the endogenous OPN expression levels in H358, MIAPaca-2, A549, and H460 cells showed a positive correlation with their cancer cell motility (Figure 2a). These observations indicated that the endogenous OPN could promote cancer cell migration.

To test the possibility that the CM produced by cancer cells would stimulate cancer cell motility, the cell migration of H358 and MIAPaca-2 cells was independently assessed in the presence of the CM derived from H358, MIAPaca-2, A549, or H460 cells (Figure 2b). The CM derived from H358 and MIAPaca-2 cells had no effect on the cell migration of H358 and MIAPaca-2 cells (Figure 2c). In contrast, the CM derived from A549 and H460 cells markedly promoted the cell migration of H358 and MIAPaca-2 cells (Figure 2c). The increased cell motility of H358 and MIAPaca-2 cells was significantly inhibited by the anti-OPN function blocking antibody (Figure 2d). These results suggest that the endogenous OPN secreted from A549 and H460 cells induced cell migration.

**Figure 3:**
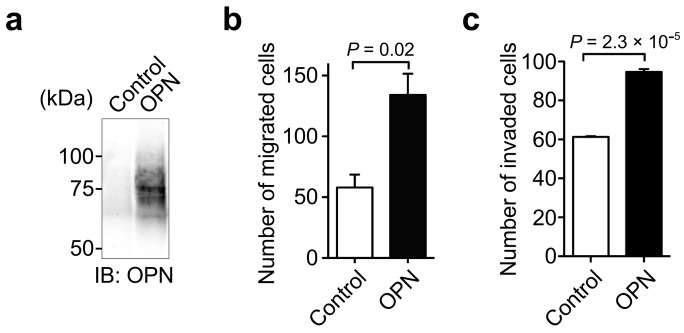
Effect of the forced expression of OPN in H358 cells on cell migration and invasion. (**a**) Expression of OPN in H358 transfectants. H358 cells with little OPN expression were transfected with either a control vector or OPN expression vector. The expression of OPN in the transfectants was confirmed by Western blot using anti-OPN polyclonal antibody (O-17). Unprocessed blot image is shown in Figure S2. Effect of OPN expression in H358 cells on cell migration (**b**) and invasion (**c**). Two-tailed unpaired Student’s *t*-test, mean ± SEM of three independent assays conducted in triplicate.

To further examine the effect of OPN on cancer cell motility, we prepared OPN stable transfectants in H358 cells (Figure 3a). Compared with the control cells, OPN-overexpressing H358 transfectants exhibited increased cell migration (Figure 3b) and invasion (Figure 3c). These results revealed a critical role of OPN in cancer cell migration and invasion.

### 3.3. OPN Phosphorylation in Cancer Cells

**Figure 4:**
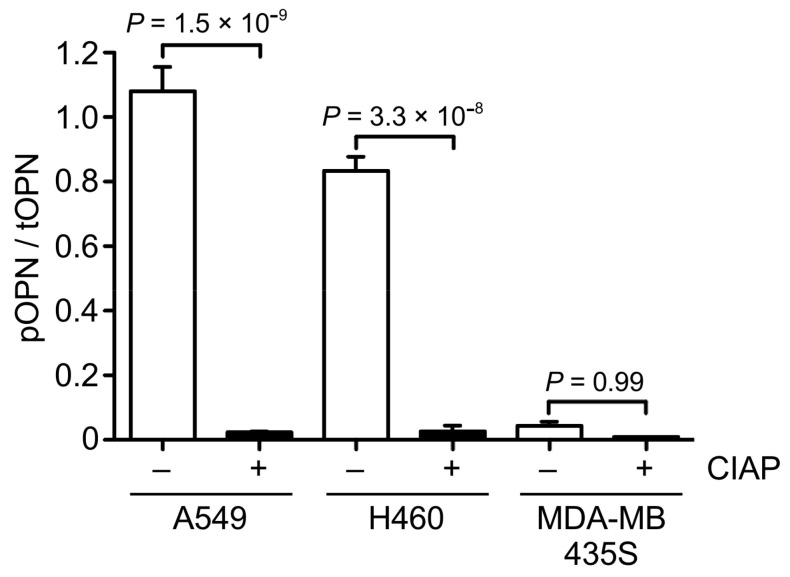
Quantitative analysis of the ratio of phosphorylated to total OPN in the CM of cancer cells. The CM of A549, H460, and MDA-MB435S cancer cells were treated with or without calf intestine alkaline phosphatase (CIAP) and were then subjected to phos-tag ELISA. Two-tailed unpaired Student’s *t*-test, mean ± SEM of three independent assays conducted in triplicate.

In general, phosphorylation is associated with dynamic changes in protein functions. Although OPN is one of the proteins with the largest proportion of phosphorylation sites [4], the relationship between phosphorylation and cancer cell migration still remains unclear. To clarify it, we first examined the ratio of phosphorylated OPN to total OPN in the CM of A549, H460, and MDA-MB435S cells by phos-tag ELISA (Figure 4). The ratio of phosphorylated OPN to total OPN in the CM of A549 and H460 cells was significantly higher than that in the A549 and H460 cells-derived CM with phosphatase treatment. On the contrary, phosphatase treatment caused no change in the ratio of phosphorylated OPN to total OPN in the CM of MDA-MB435S cells. These results suggest that phosphorylated OPN was abundant in the CM of A549 and H460 cells, whereas the CM of MDA-MB435S cells contained less phosphorylated forms of OPN.

### 3.4. Effect of OPN Phosphorylation on OPN-Induced Cancer Cell Motility

**Figure 5:**
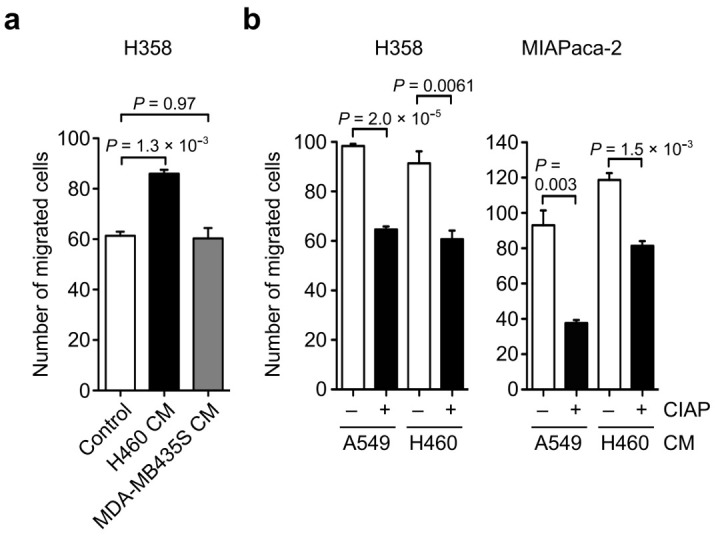
Relationship between OPN phosphorylation and OPN-induced cancer cell migration. (**a**) Effect of H460 CM and MDA-MB435S CM on cell migration of H358 cells. PBS was used as a sample instead of CM in the control experiments. One-way ANOVA and Tukey post-hoc test, mean ± SEM of three independent assays conducted in triplicate. (**b**) Effect of treatment of A549 and H460 CM with phosphatase (CIAP) on the A549 and H460 CM-induced cell migration in H358 and MIAPaca-2 cells. – and + indicate CM without and with CIAP treatment, respectively. Two-tailed unpaired Student’s *t*-test, mean ± SEM of three independent assays conducted in triplicate.

Next, we tested the effect of OPN phosphorylation on OPN-induced cell motility. The addition of H460 CM containing highly phosphorylated OPN markedly enhanced H358 cell motility compared with the control (Figure 5a). In contrast, the addition of MDA-MB435S CM containing less phosphorylated OPN had no effect on H358 cell motility. We further examined the effect of phosphatase treatment for A549 and H460 CM on A549 and H460 CM-induced cell motility in H358 and MIAPaca-2 cells. As a result, the cell migratory activity of A549 and H460 CM toward H358 and MIAPaca-2 cells was attenuated by the phosphatase treatment of CM (Figure 5b). Because A549 and H460 CM-induced cell motility in H358 and MIAPaca-2 cells was largely dependent on OPN (Figure 2d), these results indicate that the phosphorylation of OPN affects OPN-induced cell motility.

**Figure 6:**
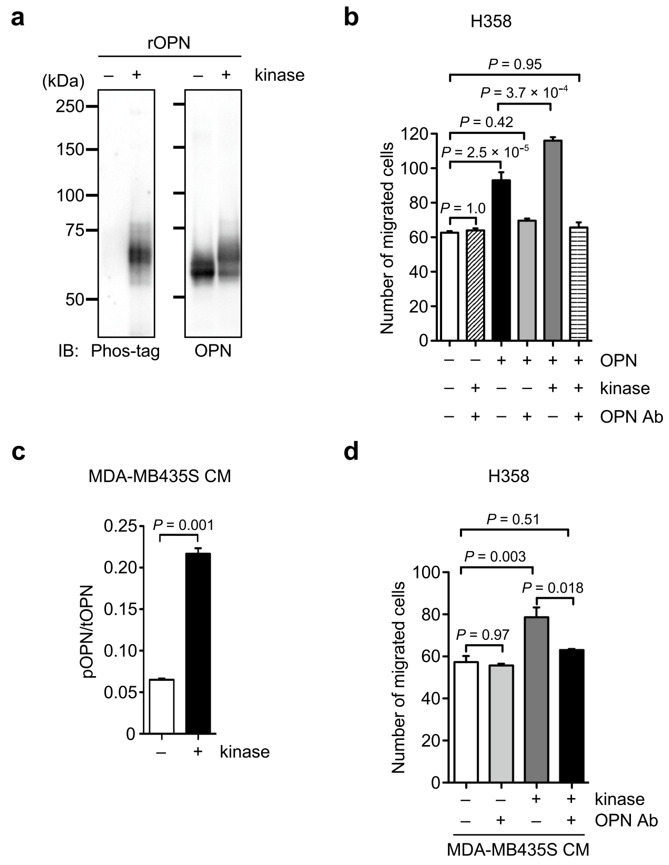
Induction of cancer cell migration by phosphorylated OPN. (**a**) Western blot analysis of phosphorylation using phos-tag in a recombinant OPN after kinase treatment. The unprocessed blot image is shown in Figure S3. (**b**) Cell migration of H358 cells in the presence or absence of recombinant OPN pretreated with kinase and OPN function-blocking antibody (OPN Ab) either separately or in combinations. One-way ANOVA and Tukey post-hoc test, mean ± SEM of three independent assays conducted in triplicate. (**c**) Phos-tag ELISA analysis of phosphorylated OPN in MDA-MB435S CM after kinase treatment. Two-tailed unpaired Student’s *t*-test, mean ± SEM of three independent assays conducted in triplicate. (**d**) Cell migration of H358 cells in the presence or absence of the MDA-MB435S CM pretreated with kinase and OPN function-blocking antibody (OPN Ab), either separately or in combination. – and + indicate treatment without and with the indicated reagent. One-way ANOVA and Tukey post-hoc test, mean ± SEM of three independent assays conducted in triplicate.

To further test the possibility that phosphorylated OPN promotes cancer cell motility, we first examined the effect of phosphorylation on OPN-induced cell motility using a recombinant OPN purified from HEK293T cells. The phosphorylation of a recombinant OPN by casein kinase II enhanced the OPN-inducing cancer cell migration, and the increased cell motility was suppressed by anti-OPN antibody (Figure 6a,b). Then, to evaluate the effect of kinase-mediated phosphorylation in cancer-secreted OPN on cancer cell migration, we performed a migration assay using MDA-MB435S CM with or without kinase treatment because the OPN in MDA-MB435S CM was less phosphorylated (Figure 4), allowing the assay to ignore the effect of endogenous phosphorylated OPN (Figure 6c,d). Similar to the results in Figure 6b, the kinase treatment of MDA-MB435S CM containing OPN with a low phosphorylation level significantly enhanced H358 cell migration compared with the control MDA-MB435S CM without kinase treatment (Figure 6d). The increased cell motility from the kinase treatment was abrogated by the anti-OPN antibody, suggesting that the upregulation of motility was due to the phosphorylated OPN in MDA-MB435S CM (Figure 6d). Taken together, these results demonstrate that the phosphorylation of OPN is related to the promotion of cancer cell motility.

## 4. Discussion

In this study, we found that MDA-MB435S, A549, and H460 cancer cell lines highly expressed OPN in both mRNA and protein levels, whereas the OPN expression levels in H358, MIAPaca-2, and Panc-1 cancer cell lines were quite low or were not detected. The OPN-expressing cancer cell lines showed higher cell migration compared with the cancer cell lines with a low OPN expression. We also demonstrated that phosphorylated OPN was abundant in A549 and H460 cells, but not in MDA-MB435S cells. Moreover, OPN phosphorylation was associated with the upregulation of OPN-induced cancer cell motility. These results indicate that phosphorylated OPN regulates cancer cell motility.

In the present study, we found a much amount of highly phosphorylated OPN in the CM of A549 and H460 cells. The ATP concentration in the extracellular environment of solid tumors was in the range of 100–500 µM, while the usual concentration of this nucleotide in the interstitium of healthy tissues is 10–100 nM [29]. In addition, extracellular phosphoproteins are particularly abundant in samples from cancer patients compared with those from healthy patients [4,7]. In fact, some phosphorylated OPNs have been identified from tumor tissues [30,31]. Therefore, it is likely that phosphorylated OPN is rich in some tumor tissues. In the tumor microenvironment, OPN is produced by macrophages and stromal cells, as well as cancer cells [12]. Thus, cancer cell migration and invasion could also be promoted by such host-cell derived phosphorylated OPN.

As depicted in Figure 2d and Figure 5b, the effect of the OPN blocking antibody and phosphatase treatment on CM-mediated migration was different in the two tested cell lines, reflecting an altered susceptibility to OPN, possibly due to the different receptor expression (integrins and CD44) and cellular signaling in H358 and MIAPaca-2 cells. Moreover, we cannot exclude the possibility that other proteins in the CM could also affect cell migration. A further comprehensive study using a phosphorylated OPN purified from cancer cells is required in order to understand the molecular mechanism of phosphorylated OPN-mediated cellular signaling and the resulting cancer cell migration.

OPN is a well-described substrate for Golgi-casein kinase FAM20C. FAM20C is widely expressed across many types of cancers compared with the adjacent normal tissues. Moreover, the FAM20C expression is positively associated with a poor prognosis in breast, lung, and colorectal cancers, as well as glioblastoma [32,33]. Our results have shown that A549 and H460 cells abundantly produced phosphorylated OPN. It is therefore possible that the OPN secreted from A549 and H460 cells may be phosphorylated by FAM20C. However, in *ras*-transformed fibroblasts and *ras*-transformed mammary cell lines, OPN is less phosphorylated along with a reduced expression of FAM20C compared with their non-transformed cells [34]. In addition, the rescue of the FAM20C expression in the *ras*-transformed fibroblasts with FAM20C transfection did not recover the phosphorylation of OPN [34], although the FAM20C expression in HEK293T cells could phosphorylate OPN [27]. These results suggest that FAM20C-meditated phosphorylation on OPN is dependent on cell types. Currently, we have no evidence that FAM20C always phosphorylates OPN in tumor tissues and cancer cells. Therefore, a further comprehensive study is required to understand the functional regulation of OPN by FAM20C-mediated phosphorylation in cancer.

In the present study, OPN was less phosphorylated in the CM of MDA-MB435S cells. However, the broad band of OPN in the CM derived from MDA-MB435S cells was observed in the Western blot analysis. As OPN is post-translationally modified with phosphorylation, glycosylation, sulfation, and transglutaminase-mediated cross-linking [35], the OPN in the CM of MDA-MB435S cells might be modified with the other post-translational modification rather than phosphorylation. Our previous studies have shown that OPN phosphorylation levels and sites are influenced by the *O*-glycosylation status [16,19]. Therefore, it is possible that the *O*-glycosylation on the OPN secreted from MDA-MB435S cells suppresses the phosphorylation on it.

OPN is an intrinsically disordered protein and generally shows a random coil-like behavior [36,37]. Intrinsically disordered proteins have many phosphorylation sites, and their phosphorylations can induce diverse structural changes and interactions with other proteins. Moreover, sequential or combinatorial multisite phosphorylations in intrinsically disordered protein enhance the molecular response [38]. In the present study, we showed that OPN-induced cancer cell motility was proportional to the amount of phosphorylated OPN in the CM of cancer cells. Recent studies have shown that the highly phosphorylated OPN purified from FAM20C-overexpressing HEK293T cells showed a decreased cell adhesion activity compared with the OPN purified from the control HEK293T cells [27]. In addition, highly phosphorylated human milk OPN promotes the cell migration of human placenta choriocarcinoma cell lines [28]. These results might reflect the effect of multisite phosphorylations on OPN functions. In contrast, signaling can also be regulated by site-specific phosphorylation in some intrinsically disordered proteins [39,40]. As site-specific phosphorylation in OPN peptides affects the binding ability of the peptides to the cells [27,35], some biological activities of phosphorylated OPN might be dependent on the phosphorylation at a specific site that is associated with OPN functions.

In conclusion, our results reveal a key role of OPN phosphorylation in regulating cancer cell migration. Thus, OPN phosphorylation may be a potential biomarker and therapeutic target for cancer.

## Supplementary Materials

The following are available online at https://www.mdpi.com/article/10.3390/biom11091323/s1, Figure S1: Unprocessed blot image in Figure 1b. Red square indicates cropped section, Figure S2: Unprocessed blot image in Figure 3a. Red square indicates cropped section, Figure S3: Unprocessed blot image in Figure 6a. Red square indicates cropped section.
