# Supplementary material for: Phosphorylated Osteopontin Secreted from Cancer Cells Induces Cancer Cell Motility
Source: Biomolecules. 2021 Sep 7;11(9):1323. doi: 10.3390/biom11091323 (PMC8470647; doi:10.3390/biom11091323)
Supplement: Supplementary file 1 [file biomolecules-11-01323-s001.zip › biomolecules-1297533-supplementary.pdf]

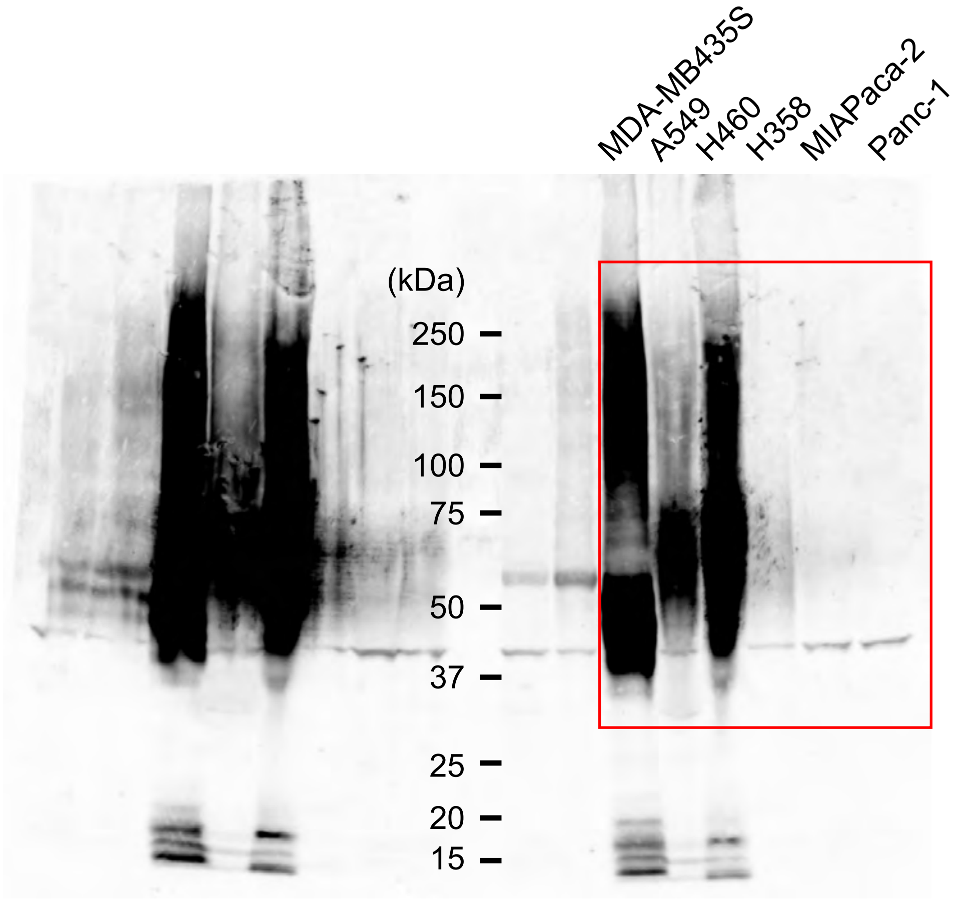

Supplementary Figure S1. Unprocessed blot image in Fig. 1b.
Red square indicates cropped section.

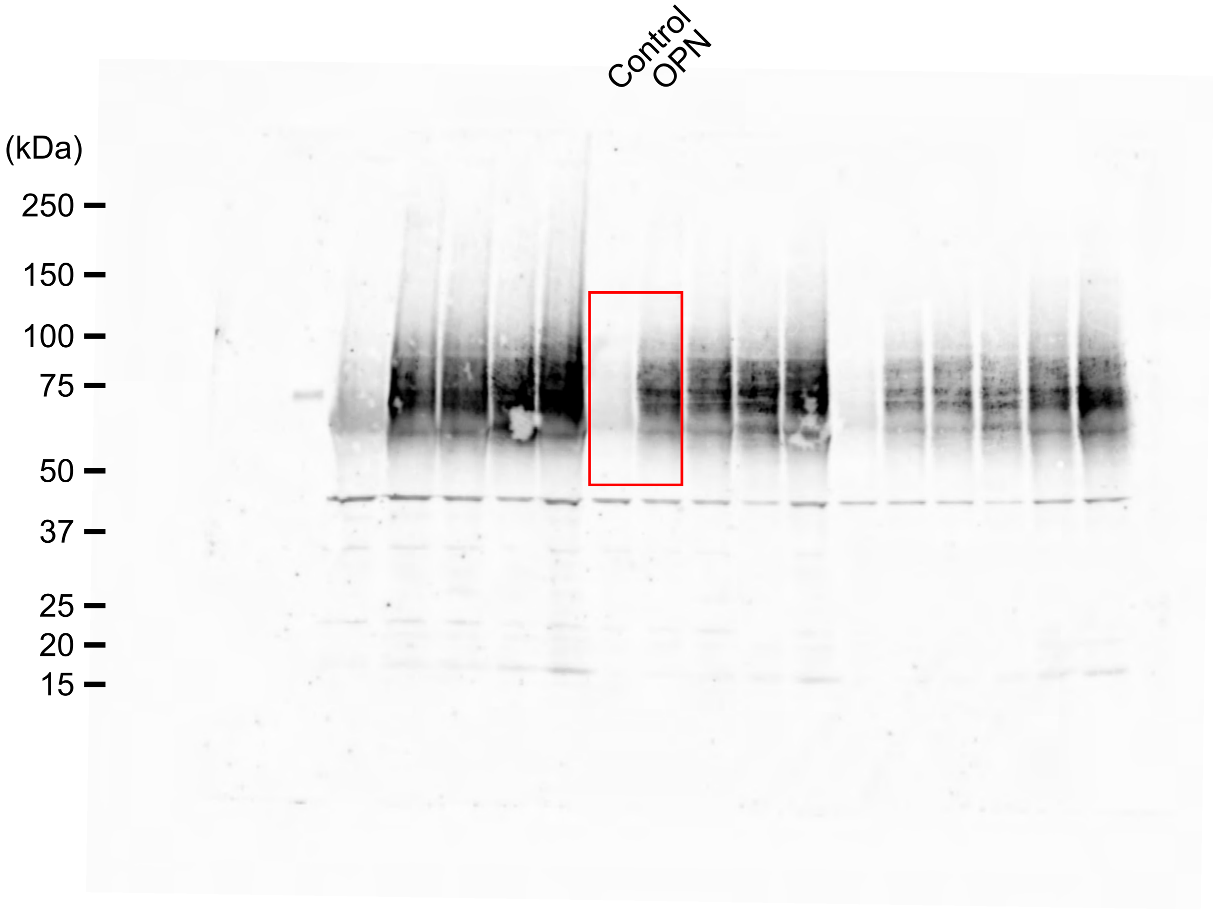

Supplementary Figure S2. Unprocessed blot image in Fig. 3a.
Red square indicates cropped section.

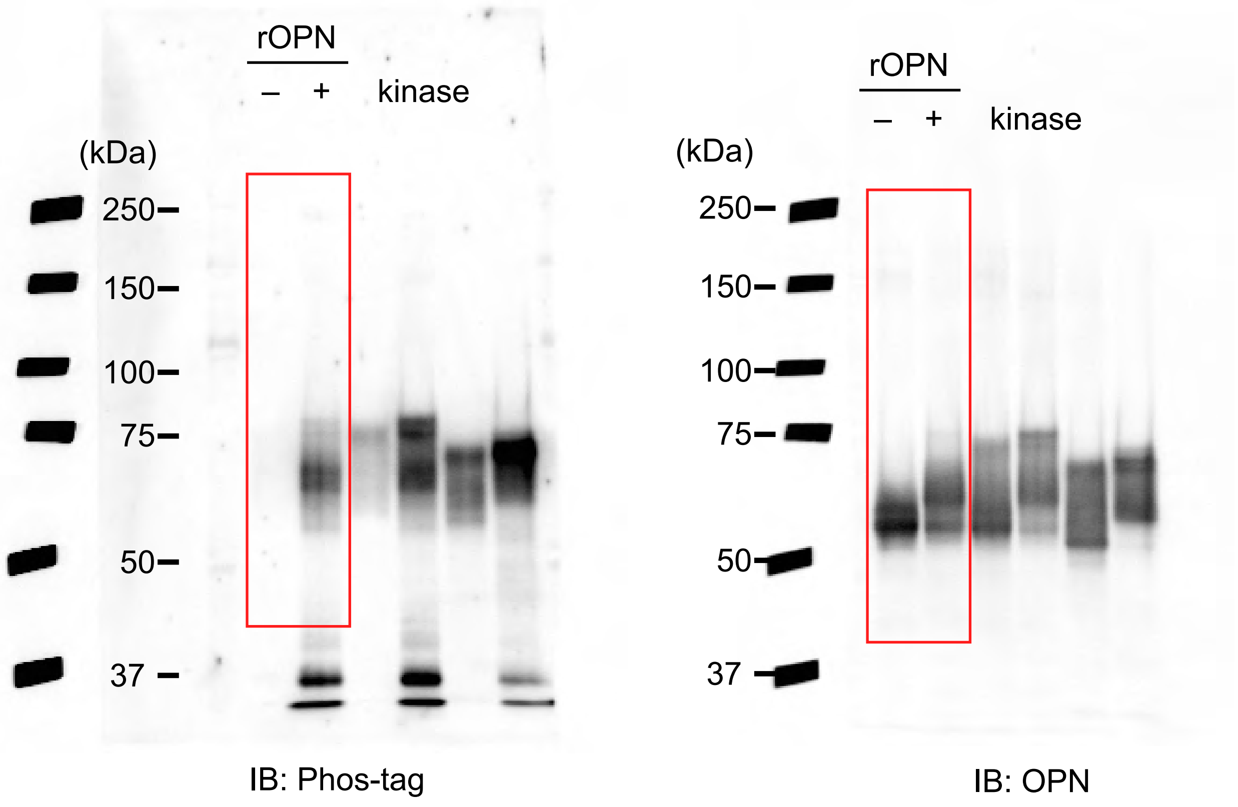

Supplementary Figure S3. Unprocessed blot image in Fig. 6a.
Red square indicates cropped section.
